# Frontal Pole Neuromodulation for Impulsivity and Suicidality in Veterans With Mild Traumatic Brain Injury and Common Co-Occurring Mental Health Conditions: Protocol for a Pilot Randomized Controlled Trial

**DOI:** 10.2196/58206

**Published:** 2024-12-13

**Authors:** Alyssa Bernanke, Rebecca Hasley, Niki Sabetfakhri, Harriet de Wit, Bridget M Smith, Lei Wang, Lisa A Brenner, Colleen Hanlon, Noah S Philip, Olusola Ajilore, Amy Herrold, Alexandra Aaronson

**Affiliations:** 1 Northwestern University Feinberg School of Medicine Chicago, IL United States; 2 Edward Hines Department of Veteran Affairs Mental Health Service Line Hines, IL United States; 3 University of Illinois, Chicago Chicago, IL United States; 4 University of Chicago Chicago, IL United States; 5 SCI/D National Program Office Veterans Health Administration Washington, DC United States; 6 The Ohio State University Columbus, OH United States; 7 University of Colorado Anschutz Medical Campus Aurora, CO United States; 8 BrainsWay Burlington, MA United States; 9 Center for Neurorestoration and Neurotechnology VA Providence Healthcare System Providence, RI United States

**Keywords:** mild traumatic brain injury, transcranial magnetic stimulation, intermittent theta burst stimulation, suicidality, suicidal ideation, impulsivity, neuromodulation, social and occupational functioning

## Abstract

**Background:**

Suicide remains a leading cause of death among veterans in the United States, and mild traumatic brain injury (mTBI) increases the risk of suicidal ideation (SI) and suicide attempts (SAs). mTBI worsens impulsivity and contributes to poor social and occupational functioning, which further increases the risk of SI and SAs. Repetitive transcranial magnetic stimulation is a neuromodulatory treatment approach that induces neuroplasticity, potentially repairing neurodamage. Intermittent theta burst stimulation (iTBS) is a second-generation form of transcranial magnetic stimulation that is safe, shorter in duration, displays a minimal side effect profile and is a promising treatment approach for impulsivity in mTBI. Our novel proposed treatment protocol uses frontal pole iTBS to target the ventromedial prefrontal cortex, which may reduce impulsivity by strengthening functional connectivity between the limbic system and frontal cortex, allowing for improved top-down control of impulsive reactions, including SI and SAs.

**Objective:**

The objectives of this study are to (1) develop an iTBS intervention for veterans with mTBI, impulsivity, and SI; (2) assess the feasibility and tolerability of the intervention; and (3) gather preliminary clinical outcome data on SI, impulsivity, and functions that will guide future studies.

**Methods:**

This is a pilot, double-blinded, randomized controlled trial. In developing this protocol, we referenced the SPIRIT (Standard Protocol Items: Recommendations for Interventional Trials) guidelines. We will enroll 56 participants (28 active iTBS and 28 sham iTBS). The iTBS intervention will be performed daily, 5 days a week, for 2 weeks. We will collect 10 validated, psychometric, quantitative outcome measures before, during, and after the intervention. Measures included will assess functioning, impulsivity, suicidality, posttraumatic stress disorder, and depressive symptoms. We will collect qualitative data through semistructured interviews to elicit feedback on the participants’ experiences and symptoms. We will perform quantitative and qualitative analyses to (1) assess the feasibility, tolerability, and acceptability of the treatment; (2) gather advanced neuroimaging data to assess neural changes elicited by treatment; and (3) assess improvements in outcome measures of impulsivity and suicidality in veterans with mTBI.

**Results:**

This study protocol was approved by the Edward Hines, Jr. VA Hospital Institutional Review Board (Hines IRB number 14-003). This novel treatment is a 5-year research project (April 1, 2023, to March 31, 2028) funded by the Veterans Administration Rehabilitation Research and Development service (CDA2 award IK2 RX002938). Study results will be disseminated at or before the project’s end date in March 2028.

**Conclusions:**

We will provide preliminary evidence of the safety, feasibility, and acceptability of a novel frontal pole iTBS treatment for mTBI, impulsivity, SI and SAs, and functional deficits.

**Trial Registration:**

ClinicalTrials.gov NCT05647044; https://clinicaltrials.gov/study/NCT05647044

**International Registered Report Identifier (IRRID):**

PRR1-10.2196/58206

## Introduction

### Background

The prevalence of suicide among veterans who served in the US military is devastatingly high. Veterans die by suicide at almost double the rate of civilians in the United States [1]. The prevalence of suicide among veterans with traumatic brain injury (TBI) is even higher. Veterans with TBI are 1.5 times more likely to die by suicide than veterans without TBI, even when controlling for comorbid psychiatric conditions [1,2]. These statistics are particularly concerning given that there have been >400,000 new TBI diagnoses among US service members since the beginning of operations Enduring Freedom, Iraqi Freedom, and New Dawn, of which >80% are mild traumatic brain injury (mTBI) [3]. Overall, between 16% and 20% of all operations Enduring Freedom, Iraqi Freedom, and New Dawn veterans have a history of mTBI, and among them, up to 22% report suicidal ideation (SI) [4].

TBI consists of any external force applied to the head of any individual, resulting in vascular and axonal damage, edema, and neuronal death [5]. TBI is clinically further subcategorized by the severity of the injury as either mild, moderate, or severe. mTBI is defined as a loss of consciousness for <30 minutes, postconcussive altered mental status or amnesia for <24 hours, a Glasgow Coma Score, following injury of 13 to 15 (15 being the maximum score), and lack of abnormalities on standard clinical magnetic resonance imaging (MRI) or computed tomography [6]. TBI is an extremely common injury, resulting in an estimated 4.8 million independent emergency room visits throughout the United States annually [7-9]. TBI results in a significant economic burden: the cost of nonfatal TBI amounts to >US $40 billion per year in the United States [10]. Globally, TBI resulted in the loss of 246.7 million disability-adjusted life years between 1990 and 2013 [11].

Given the high suicide rate among US veterans, suicide prevention is a high-priority issue for the US Veterans Administration (VA) [12]. The US VA has assembled task forces that have created clinical practice guidelines for the assessment and management of veterans at risk for suicide [13]. These guidelines establish substantial risk factors to help providers identify which veterans are at the highest risk. The guidelines specifically cite having a history of TBI, functional deficits, past SI, and a history of impulsivity as independent risk factors for developing SI [13]. TBI predisposes individuals to functional deficits [14,15] and impulsivity [16], and those with impulsivity likely struggle with functional deficits [17]. Given these findings, we hypothesize that these conditions may be interrelated and could serve as meaningful behavioral targets in the treatment of SI.

Transcranial magnetic stimulation (TMS) therapy was first described by Barker et al [18]. Seminal studies by George et al [19] demonstrated the efficacy of TMS in the treatment of depression in large, multisite sham-controlled trials. TMS was subsequently approved by the US Food and Drug Administration (FDA) in 2008 for the treatment of major depressive disorder [20]. TMS was first hypothesized as a treatment for TBI by Pape et al [21]. It was later shown to be effective in treating several TBI sequelae, including depression [22]. TMS also has demonstrated efficacy in the treatment of posttraumatic stress disorder (PTSD), chronic pain, cognitive deficits, and headaches in individuals with mTBI [23-27]. A second-generation form of TMS, intermittent theta burst stimulation (iTBS), was first shown to treat depression in 2009 in a study by Holzer and Padberg [28]. In a large, noninferiority study comparing iTBS and TMS, patients with treatment-resistant depression show similar safety and adverse event (AE) profiles and exhibit improvements in their depressive symptoms to both interventions [29].

This study aims to create a novel iTBS intervention to reduce impulsivity and SI among veterans with mTBI while improving overall social and occupational functioning. This study will be conducted at the Edward Hines, Jr. VA Hospital in Hines, Illinois. We are interested in stimulating the ventromedial prefrontal cortex (VMPFC) with iTBS because the VMPFC appears to exhibit less functional connectivity with the limbic system in individuals with mTBI [30]. This diminished connectivity is suspected to be responsible for impulsivity and SI in individuals with mTBI [30-32]. To the best of our knowledge, no established treatment approach has used VMPFC iTBS to improve social and occupational functioning, SI, and negative urgency impulsivity among individuals with mTBI and comorbid mental illness. A list of the common terms used in the study and their definitions are provided in Textbox 1.

**Terms and definitions used in the study.**

- Mild traumatic brain injury: a type of brain injury resulting from mechanical forces defined by loss of consciousness for <30 minutes, Glasgow Coma Score of 13-15, post-concussive altered mental status or amnesia <24 hours, and normal findings on neuroimaging
- Negative urgency impulsivity: a form of impulsivity in which an individual exhibits poor behavioral control or aggression in the setting of a negative event or affect
- Neuromodulation: a category of brain treatments that modulate or alter neuronal activity through direct electrical or chemical stimulation
- Transcranial magnetic stimulation: a non-invasive brain treatment that applies a magnetic field directly to the scalp, activating neuronal activity and neuroplasticity to specific cortical regions of the brain
- Intermittent theta burst stimulation: a novel second-generation form of transcranial magnetic stimulation that utilizes bursts of magnetic energy known as theta bursts

### Rationale

#### SI Epidemiology and Current Treatment

SI is a severe and prevalent condition among veterans and civilians alike and can prove lethal. The Centers for Disease Control and Prevention estimates that from 2015 to 2019, 4.3% of the general adult US population had SI, and 5% to 10% of these individuals advanced to making a suicide attempt (SA) [33]. Consequently, focusing treatment on individuals with SI before they advance to making an SA gives providers an opportunity at suicide prevention.

Current evidence-based treatment options for SI include pharmacotherapy and psychotherapy. Pharmacotherapeutic treatment (primarily antidepressants) can take 6 to 8 weeks or more to become effective [34]. A recent meta-analysis suggested that only two-thirds of the published studies demonstrated a reduction in SI with any pharmacotherapy compared to placebo [35]. Furthermore, many patients will require multiple medication trials and may take months to experience any symptomatic improvement [36]. Given the latency of these treatment options, individuals with SI require more suicide prevention interventions that are designed to exhibit better and faster resolutions toward promoting resilience.

#### mTBI and Suicide: The Role of Impulsivity

Impulsivity is associated with TBI of all severities [16,37,38]. For this study, we will focus on veterans with mTBI. Veterans with mTBI make up the majority (>80%) of the veteran population with TBI in the United States [3]. A form of behavioral impulsivity, negative urgency impulsivity, is particularly prevalent in this population [38]. Negative urgency impulsivity refers to poor behavioral control in the setting of negative events or affect [39]. It is associated with excessive agitation, irritability, low moods, and a greater tendency for aggression toward oneself or others immediately following a negative event [40]. Unsurprisingly, negative urgency impulsivity increases the likelihood of SI [41] and is associated with significant risk factors for suicide, such as social and occupational functional deficits [42]. Our laboratory’s previous work built upon these findings and completed a chart review study that found negative urgency impulsivity most strongly mediated the relationship between a history of TBI and SI and SAs [43].

**Figure 1:**
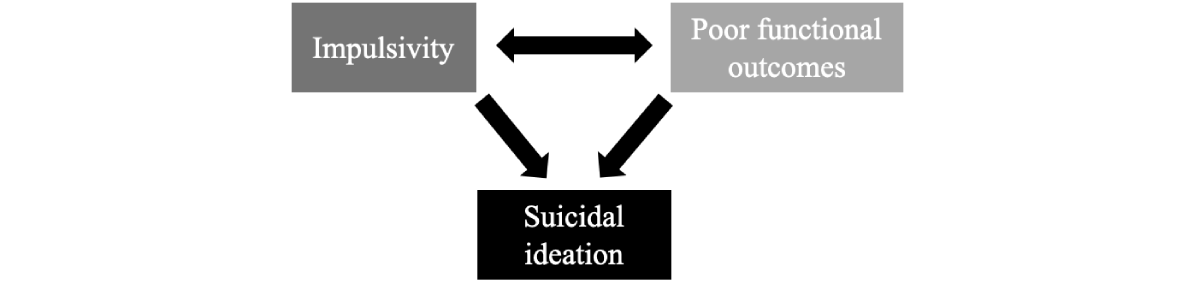
Relationship between impulsivity, functionality outcomes, and suicidal ideation.

Post-TBI negative urgency impulsivity is presumed to be secondary to disinhibition of the limbic system from TBI-related damage to the prefrontal cortex, specifically the VMPFC, medial orbitofrontal cortex, or both [16]. The VMPFC, which serves as an inhibitory control center for the limbic system, is particularly vulnerable to damage in all severities of TBI as this region abuts the cranial floor and frontal cranial bone, making it susceptible to coup and contrecoup forces from nearly all directions [31,44,45]. Uninhibited limbic effect can manifest as poor frustration tolerance, fear, and aggression [46]. These characteristics of negative urgency impulsivity can reduce the quality of life and worsen community and social functioning, predisposing individuals to SI and SAs (Figure 1) [46].

To the best of our knowledge, no neuroimaging evidence implicates specific brain regions in mTBI-related impulsivity and SI. The presented study will help address this knowledge gap using within-participant neuroimaging data to identify brain regions predisposed to these conditions.

#### mTBI and Suicide: The Role of Functional Deficits

TBI itself predisposes individuals to social and occupational functional deficits [47]. A meta-analysis of psychosocial factors found that difficulties in social and occupational functioning were strongly tied to increased levels of SI, social isolation, and low socioeconomic status and are directly associated with increased incidences of SI and completed suicides (Figure 1) [48]. Among individuals with TBI, those who were unemployed in the past year were at the highest risk for SI when compared to individuals who had been employed [17]. Given that TBI and impulsivity are both (1) independent risk factors for developing SI and (2) associated with impaired social and community functioning, it appears that TBI, SI, impulsivity, and functional deficits are intertwined and should be considered in tandem. However, the evidence is scarce to directly support biological treatments for SI, impulsivity, and functional deficits following mTBI. This study aims to address this knowledge gap by providing further evidence to support the development of a novel neuromodulatory treatment to improve functioning for those with impulsivity, SI, and mTBI symptoms.

#### TMS in Neuropsychiatry

TMS is a noninvasive neuromodulatory technique that involves placing an insulated electric coil on the scalp [49]. The electrical current flowing through the coil produces alternating magnetic fields. These fields pass through the skull, inducing electric currents in the brain tissue beneath the coil [50]. In large, multisite, double-blinded, sham-controlled trials, TMS applied [51,52] to the left dorsolateral prefrontal cortex (DLPFC) was shown to effectively treat depression. TMS was subsequently approved by the FDA to treat treatment-resistant depression in 2008 [53-55]. In 2020, a nationwide clinical TMS program was implemented in the US VA after completing a multisite clinical trial. Thus, the VA health care system is well-positioned to offer novel TMS treatment approaches for veterans in need.

iTBS is a second-generation FDA-approved form of TMS designed to mimic endogenous brain rhythms with the added benefit of markedly reduced treatment time [28,56]. In iTBS, 50 Hz triplets are applied at a theta rhythm (5 Hz), which is thought to facilitate long-term potentiation in less time and with fewer pulses than standard TMS, which generally is administered at 5 to 10 Hz [57]. Using this approach, iTBS can be administered in 3 to 10 minutes, compared to the standard 37.5-minute protocol for first-generation TMS [58]. One of the most prevalent and widely known iTBS treatment protocols is based on a study by Li et al [58] and delivers 1800 pulses of iTBS per day for 10 days. This amount of stimulation appears to be well tolerated and shows a good effect on depressive symptoms, even in a treatment-refractory group [58]. Studies demonstrate that this number of pulses results in an optimal “window” of cortical excitability, and additional pulses may have an inhibitory effect [59-61]. The result of significant symptomatic improvement in a short period is particularly interesting and relevant in treating SI, where a shorter latency to treatment response is of the essence and could potentially be lifesaving.

#### TMS as a Treatment for TBI Sequelae

TMS has an established safety record as a treatment for individuals with mTBI [23]. There is accumulating evidence supporting the safety and efficacy of TMS for sequelae of mTBI and related neuropsychiatric conditions, including depression, cognitive deficits, and chronic pain or headaches [23-26]. TMS also improves PTSD symptoms among those with co-occurring mTBI [22,27]. Further, multiple studies and meta-analyses demonstrate that TMS, administered to a variety of brain locations, including the DLPFC, does not induce more significant numbers of AEs among individuals with mTBI than among individuals without mTBI [23,25,62,63]. Specifically, in a national US VA TMS study of 770 veterans receiving TMS, nearly half had mTBI, and TMS was demonstrated to be equally safe in veterans with and without mTBI [64].

An overall estimate of 70% of individuals with mTBI struggle with at least one psychiatric comorbidity [65]. Thus, it is essential to consider TBI and comorbid mental illness simultaneously, as they frequently co-occur. Comorbid conditions include substance use disorders, PTSD, and depression [66-68]. Moreover, neuroimaging studies of individuals diagnosed with PTSD and substance use disorder (2 conditions that are also associated with impulsive behavior and SI) demonstrate diminished volume and hypoactivity in the VMPFC [69,70]. Therefore, it is likely that when mental illness diagnoses such as PTSD and substance use disorders co-occur with mTBI, they have an additive effect on brain functioning in the VMPFC, further worsening negative urgency impulsivity [69,70].

#### TMS as Treatment for Suicidality

Encouraging meta-analyses suggest that TMS may also effectively treat suicidality [71,72]. For example, a randomized controlled trial (RCT) reported a 44% decrease in individuals experiencing “thoughts of suicide” after high-dose left-sided DLPFC TMS treatment [73]. In addition, a study of pooled sham-controlled trials reported that bilateral TMS is associated with a decline in SI [74].

However, drawing overall conclusions from the existing literature on TMS for suicidality remains challenging because of the heterogeneity of the data. Studies are often limited by small sample sizes, inconsistencies in how suicidality is measured across studies, and contradictory findings [75,76]. Interestingly, an analysis suggests that changes in SI may be statistically independent of depression outcomes, underscoring the importance of studying SI separately from depression in contrast to many previous studies [77].

Post-TBI negative urgency impulsivity is presumed to arise from limbic system disinhibition because of damage to the VMPFC [16]. In mTBI, thinning of the right VMPFC is evident when compared to healthy controls [78]. Individuals exhibiting this volume loss report increased aggression, anxiety, and depression following injury [79]. Uninhibited limbic effect can manifest as poor frustration tolerance and aggression, characteristics of negative urgency impulsivity [80]. Interestingly, individuals without a history of mTBI who attempted suicide or have a history of SI also show diminished VMPFC volume and reduced connectivity between the prefrontal cortex and the limbic system, further supporting it as a potential therapeutic target [31,81]. Our study will collect MRI data to support our hypothesis that iTBS increases neuroconnectivity between the VMPFC and limbic system.

Frontal pole stimulation is a recent TMS and iTBS site of interest for reducing impulsive behaviors, including substance abuse [82] and compulsions in obsessive compulsive disorder [83]. iTBS can be applied to the frontal pole to target the VMPFC, and iTBS to the frontal pole is tolerable, with no more subjective pain than TMS [63]. This approach allows for more direct stimulation of the VMPFC and modulation of the limbic system [63,82].

This study proposes inducing neuroplasticity to the VMPFC by stimulating the frontal pole to treat SI and impulsivity in veterans with mTBI. We selected the frontal pole and associated right VMPFC as a treatment target as volumetric data demonstrates thinning of the right medial orbitofrontal cortex and VMPFC when compared to healthy controls [78]. Individuals exhibiting this volume loss also report increased aggression, anxiety, and depression following injury [79]. The role of the VMPFC in negative urgency impulsivity is also supported by evidence that individuals with penetrating TBI to the VMPFC are significantly more likely to become aggressive than those with penetrating TBI elsewhere [44]. By inducing modulation and neuroplasticity to the VMPFC and limbic system, iTBS at the frontal pole offers a promising means of mitigating impulsive behaviors and decreasing SI by increasing connectivity and undoing limbic damage caused by mTBI. However, more research is essential to ensure safety, tolerability, and efficacy of this treatment approach.

### Objectives

This pilot study aimed to (1) determine the safety, feasibility, tolerability, and efficacy of frontal pole iTBS for veterans with mTBI, negative urgency impulsivity, and SI; (2) collect advanced neuroimaging data from veterans with mTBI, impulsivity, and suicidality to further our understanding of what neural changes are occurring when treatment is effective; and (3) gather preliminary clinical outcome data on impulsivity, SI, and social and occupational functioning of veterans receiving iTBS. The results of these objectives will help to guide future studies on neuromodulation as a treatment for impulsivity and SI among patients with mTBI.

### Hypotheses

Our central hypotheses are that frontal pole iTBS will be safe, feasible, and tolerable among veterans with mTBI based on existing literature that suggests frontal pole iTBS is as safe and tolerable as iTBS performed elsewhere [23,25,62]. Similarly, iTBS, when administered to individuals with mTBI is as safe and tolerable as in individuals without mTBI [63]. Given that we are using an established iTBS protocol within the established TMS safety guidelines [84], we do not anticipate significant AEs.

We further hypothesize that social and occupational functioning, negative urgency impulsivity, and SI will be improved for individuals who receive active-iTBS compared with those who receive sham-iTBS. Even if only a single domain improves, we consider this a positive finding.

In addition, we hypothesize that resting-state functional connectivity between the VMPFC and the limbic system (especially the amygdala and anterior cingulate cortex) will be strengthened for the active-iTBS group compared with the sham-iTBS group. We also expect that veterans with increased connectivity between these regions of interest (ROIs) will likely show the most functional improvements.

Pending the results of this pilot trial, larger-scale RCTs may be warranted to establish the efficacy of frontal pole iTBS for social and occupational functional deficits, negative urgency impulsivity, and SI.

## Methods

### Study Design

The proposed study is a pilot, prospective, randomized, double-blinded, sham-controlled study to develop a frontal pole iTBS intervention. We will examine the safety, feasibility, tolerability, and efficacy of 10 sessions of iTBS over 2 weeks. Preliminary impulsivity, SI, and social and occupational functioning data will be collected before and after receiving active or sham treatment. In developing this protocol, we referenced the SPIRIT (Standard Protocol Items: Recommendations for Interventional Trials) guidelines [85]. We used the CONSORT (Consolidated Standards of Reporting Trials) checklist when writing our report [86]. Refer to Multimedia Appendix 1 for the CONSORT flow diagram.

### Ethical Considerations

The study protocol was reviewed and approved by the Edward Hines, Jr. VA Hospital Institutional Review Board (Hines IRB number 1716074) and the University of Illinois, Chicago Institutional Review Board. The study is registered on ClinicalTrials.gov (NCT05647044). This study is designed in accordance with the ethical standards of the US VA Office of Research and Development, based on those from the Declaration of Helsinki, the Belmont Report, US Common Rule and Regulations from the Council for International Organizations of Medical Sciences. The study will be overseen by the institutional review boards of the both Edward Hines, Jr. VA Hospital and the University of Illinois, Chicago. This research study is designed on a 5-year research plan.

A Health Insurance Portability and Accountability Act (HIPAA) authorization waiver will be obtained to enable electronic medical records review of potential participants before obtaining consent. The HIPAA waiver allows research staff to access information about the patient’s medical history and demographics, as well as history of drug or alcohol abuse to determine study eligibility. If a potential participant declines to participate in the study, no further contact will be made.

If the potential participant consents to the study, they will then begin the informed consent process. Informed consent will be obtained in English by trained research staff. Research participants will have access to research staff and the principal investigator to assist with any questions or concerns until understanding is achieved. A consent note is made in the participant’s medical record either electronically or on paper. The original consent is then placed in the medical record, a copy is given to the participant, a copy is filed with the Edward Hines, Jr. VA Hospital Institutional Review Board and the original informed consent is placed in the participant’s research binder and maintained in a locked file cabinet at Hines VA Hospital. Once the informed consent process is completed, a note will be documented in the electronic medical record system and the participant will be flagged as a research participant.

Any identifiable information about the participant, including their signed consent form, is stored in a different location than study data, on encrypted VA servers in files that are only accessible to approved study team members. The collected, deidentified research data are also stored on encrypted VA servers elsewhere, in password-protected folders, only accessible to approved study team personnel. Any participant information obtained in the study will be treated as confidential and safeguarded in accordance with the Privacy Act of 1974. Information published or presented about the results of the study will be done such that no individual identifying information is shared.

Participants will be compensated up to US $350 for their participation, prorated for their time.

### Participants

We will enroll 56 veterans aged between 22 and 65 years with a history of mTBI and impulsivity. They must also report that they have experienced SI within the last 3 months. mTBI will be defined according to the VA and Department of Defense guidelines [87] and use aspects of the mTBI symptom attribution and classification algorithm [88] when determining eligibility. mTBI must have occurred at least 1 year before enrollment to ensure that the injury has stabilized. Though it is not mandatory, veterans may also have diagnoses of depression, PTSD, or bipolar spectrum illness (without a history of psychosis). Recent SI (within the past 3 months) will be defined as a score of ≥1 on the Columbia Suicide Severity Rating Scale [89]. Impulsivity will be defined as having any mentions of impulsive behavior in the medical chart and any positive results on the Urgency, Premeditation (lack of), Perseveration (lack of), Sensation Seeking, Positive Urgency Impulsive Behavior Scale–Negative Urgency subscale (UPPS-P) [41]. For safety, we will exclude persons with contraindications to TMS or MRI because of increased risk of AEs. We will also exclude participants whose SI is active, with intent and plan, as they require emergency psychiatric intervention. We will not exclude individuals based on sex, gender identity, ethnicity, or race. To enhance generalizability, we will include veterans regardless of previous combat or deployment experience. As cannabis and alcohol use is prevalent among veterans, intermittent or low amounts of cannabis or alcohol consumption will not be considered exclusionary if the use does not meet the criteria for a moderate substance use disorder. Detailed information on inclusion and exclusion criteria is listed below (Textbox 2).

**Participant inclusion and exclusion criteria.**

**Inclusion criteria**

- Aged between 22 and 65 years
- Can read and speak English
- Mild traumatic brain injury (mTBI) criteria: Symptom Attribution and Classification Algorithm [88] criteria for mTBI (without the requirement of clinical neuropsychological impairment).
- Columbia Suicide Severity Rating Scale score of ≥1 within 3 months
- History of impulsivity documented in the chart and exhibited on the Urgency, Premeditation (lack of), Perseveration (lack of), Sensation Seeking, Positive Urgency Impulsive Behavior Scale—Negative Urgency subscale (Urgency score >20) [41]

**Exclusion criteria**

- Contraindications to intermittent theta burst stimulation or transcranial magnetic stimulation (eg, epilepsy)
- Contraindications to magnetic resonance imaging (eg, claustrophobia, ferromagnetic metal implants)
- Active substance use disorder per DSM-5 (Diagnostic and Statistical Manual of Mental Disorders, Fifth Edition) criteria
- Active suicidal ideation with intent and plan (these participants will be brought to the emergency department for emergent psychiatric care)
- History of moderate to severe traumatic brain injury
- History of nontraumatic neurological injury (eg, stroke, neurosurgery, hemorrhage)
- History of or current psychosis not due to an external cause (eg, due to illicit drug use)
- Pregnant or breastfeeding
- Within 12 weeks of a major surgery
- Active, unstable health condition (ie, decompensated heart failure, recent severe heart attack)
- Within 1 year of mTBI

### Power Analysis and Sample Size Justification

To ensure we have adequate power to detect a difference in our primary outcome, the Social and Occupational Functioning Assessment Scale (SOFAS), we conducted a power analysis. Assuming a significance level of 0.05, a mean SOFAS score of 44.6, and a SD of 15, with 50 veterans, we will have 82% power to detect a significant interaction between time and treatment assuming no change over time in the sham-iTBS group and a change of 13 points in the active iTBS group [90]. On the basis of similar studies of iTBS protocols for patients with depression and PTSD, receiving treatment of similar duration, attrition rates are expected to be low—under 10% [58,90,91]. As such, we plan to enroll participants to ensure we have an adequate sample size considering 10% attrition. Thus, our total sample size will be 56 with 28 in the active iTBS group and 28 in the sham iTBS group.

### Recruitment

We will recruit veterans receiving care from the TBI and polytrauma program, primary clinic, women’s health clinic, and the Mental Health Service Line at Edward Hines, Jr. VA Hospital in Hines, Illinois, United States. Personnel at all mentioned clinics will be educated on the study and encouraged to give institutional review board (IRB)–approved flyers containing contact information to veterans who may be a good fit for the study. We will be added as cosigners in the electronic medical records of Hines VA patients who may be deemed a good fit for this study. We will also mail informational letters to veterans who have completed past TBI studies and permit us to contact them about future studies through a TBI Data Repository (Hines IRB number14-003), which currently includes >300 veterans. Research candidates at the Hines VA will also be identified using administrative electronic health record data available through the VA Informatics and Computing Infrastructure. The VA Informatics and Computing Infrastructure database will search for TBI within the past 10 years (FY2013-FY2023) according to appropriate *International Classification of Diseases* (ninth and tenth editions), clinical modification, codes reflecting study inclusion criteria. All potentially eligible veterans will receive an informational letter and phone call to gauge interest and perform initial eligibility screens.

### Data Collection and Measures

#### MRI Data Acquisition, Neuronavigation, and Motor Thresholding

All participants will undergo an MRI of their brain before and after iTBS administration. Before iTBS, a high-resolution, 3D, T1-weighted, multigradient-echo sagittal anatomical scan (voxel size=0.8 mm isotropic resolution) will be collected at the University of Illinois, Chicago Center for Magnetic Resonance Research to allow for iTBS treatment site neuronavigation for each participant. Participants will also complete a resting-state functional MRI (fMRI) and diffusion tensor imaging before and after iTBS administration to examine treatment-induced connectivity changes. Participants will be asked to complete a standardized MRI safety screening form before starting the study to ensure the MRI safety procedures are followed (Multimedia Appendix 2).

Each participant’s T1 MRI will be loaded into a TMS Neural Navigator TS system (version 3.3.22, Localite), consisting of a camera and computer system that allows a TMS provider to superimpose an individual’s MRI onto where they are in space, allowing for very precise placement of the TMS coil. Electrodes will be placed on the thumb over the abductor pollicis brevis (ABP) muscle and connected to an electromyograph (MagVenture) to measure motor-evoked potentials (MEPs). To determine each participant’s motor threshold (MT) for iTBS (to determine at which intensity the iTBS stimulation should be delivered), a MagProX100 with MagOption stimulator and Magpro Cool Coil B65 A/P (MagVenture) will be used. Single pulse TMS will be administered to the left motor cortex at the hand knob. The location of this part of the motor cortex will be estimated using the participant’s MRI and then confirmed using TMS to identify the ABP muscle neural coordinates. The resting MT is the lowest stimulus intensity necessary to produce MEPs of the peak-to-peak amplitude of ≥50 µV in 5 of 10 trials. MEPs of the ABP will be recorded using surface electrodes on the right thumb. The right frontal pole will be identified and mapped using the TMS Neural Navigator (Localite) system. The anatomical location is easily identifiable using anatomical landmarks and correlates with electrode frontal polar cortex (FP; FP2) on a 10 to 20 electroencephalogram, a region overlapping with the right VMPFC [22,63,82]. Refer to Multimedia Appendix 3 for the equipment list.

#### Data Collection

Data collection is expected to take approximately 18 to 20 hours per participant over the intervention period of 2 weeks. Assessments will be collected at 4 time points in the study: screening, baseline, midpoint, and end point (Table 1).

**Table 1:** Assessment timeline and purpose.

Assessment name		Assessment purpose	Items	Scoring	Validity and reliability
**Phone screening assessments**					
	Ohio State TBI^a^ Identification Method [92] (rater administered)	mTBI^b^ eligibility determination	Structured interview	Determination of the likelihood TBI sustained based on the participant’s detail recollection	Interrater reliability >90% (very high) [92]
	Columbia Suicide Severity Rating Scale [89] (rater administered)	History of suicidality, eligibility determination—secondary outcome	Five items measuring the presence of SI^c^, intensity of SI, presence of suicidal behaviors, lethality of behaviors	Ideation: 0-5; intensity: 2-25; suicidal behaviors: yes or no; lethality: 0-2, higher scores=more severe	Internal validity a=0.95 and test-retest reliability r_*tt*_*=*0.90-1.0 (very high) [93,94]
	Urgency, Premeditation (lack of), Perseveration (lack of), Sensation Seeking, Positive Urgency Impulsive Behavior Scale—Negative Urgency subscale [41,95] (rater administered)	Negative urgency impulsivity, eligibility determination—secondary outcome	Fifty-nine items measuring 4 traits–urgency, lack of premeditation, lack of perseveration, and sensation seeking [96]	4-point Likert scale questions, scored by each trait, some reverse-coded. Higher score=more severe [97]	Cronbach α=0.91 (very high) [41,97]
	Demographics (rater administered)	Sample characterization	—^d^	—	—
	UIC^e^ CMRR^f^ MRI^g^ Safety Form (Multimedia Appendix 2; rater administered)	MRI safety, compatibility, and eligibility determination	—	—	—
	TMS^h^ Safety Form (Multimedia Appendix 4; rater administered)	TMS safety compatibility, eligibility determination	—	—	—
**In-person screening assessments**					
	Structured diagnostic interview [88] (rater administered)	mTBI symptoms, eligibility determination	Structured interview	Likelihood symptoms owing to TBI sustained	—
	Neurobehavioral Symptom Inventory [98] (rater administered)	mTBI symptoms, eligibility determination	Twenty-two items asking about the severity of postconcussive symptoms	Severity: 0=never to 4=very severe, symptom always present; higher score=more severe symptoms	Cronbach α=0.81-0.96 (very high) [98]
	Clinically Administered PTSD^i^ Scale for DSM-5^j^ [99] (rater administered)	PTSD diagnosis	30-item questionnaire corresponding to DSM-5 PTSD criteria	Severity: 0=absent to 4=extreme, total symptom severity 0-80, higher score=more severe symptoms	Interrater reliability *K*=0.78-1.0 (high) discriminant validity: rs=0.02−0.54 (moderate) [100]
	Alcohol Use Disorder Identification Test [101] (participant self-administered)	Active alcohol use disorder, eligibility determination	10-item questionnaire about the frequency and amount of alcohol used	Questions 1-8 (0-4 points), 9-10 (0,2,4 points, range of 0-40 points, higher score=more severe symptoms)	AUROC^k^=0.891 (high) [101]
	Drug Use Disorders Identification Test [102] (participant self-administered)	Active substance abuse disorder, eligibility determination	11-item questionnaire about the frequency and number of drugs of abuse used	Questions 1-9 (0-4 points), 10-11 (0,2,4 points, total score 0-44, higher score=more severe symptoms)	Cronbach α=0.93 (very high), convergent validity *r*=0.76 (high) [102,103]
	Structured Clinical Interview for DSM-5, Research Version [104] (rater administered)	Active mental illness diagnosis	Structured interview	—	*K*>0.4 (above average) sensitivity of identifying diagnosis >0.8 (good) [105]
**Baseline, midpoint, and follow-up assessments**					
	Social and Occupational Functioning Assessment Scale [106] (rater administered)	Functioning in the community–primary outcome	Single item 100-point scale assessing social activities, relationships, self-care, aggressive behavior	0-100 (0=not functioning, 100=superior functioning)	Weighted *K*=0.95 (very high) [107]
	Columbia Suicide Severity Rating Scale (rater administered)	As described in “phone screening assessments”	As described in “phone screening assessments”	As described in “phone screening assessments”	As described in “phone screening assessments”
	Urgency, Premeditation (lack of), Perseveration (lack of), Sensation Seeking, Positive Urgency Impulsive Behavior Scale—Negative Urgency subscale (participant self-administered)	As described in “phone screening assessments”	As described in “phone screening assessments”	As described in “phone screening assessments”	As described in “phone screening assessments”
	Buss-Perry Aggressiveness Scale [108] (participant self-administered)	Aggression symptoms	29-item scale of true or false statements about aggressive and stable behaviors, including measures of physical aggression, verbal aggression, anger, and hostility	Scale of 1-5, 1 being “extremely uncharacteristic,” to 5 being “extremely characteristic.” Some reverse coded (stability statements), higher score=more aggressiveness	Test-retest reliability r_*tt*_=0.80 (high), Cronbach α=0.68-0.8 for each subscale (moderate-high) [108]
	Patient Health Questionnaire-9 [109] (participant self-administered)	Depression symptoms	9-item scale of DSM-5 depressive disorder symptoms (ie, low mood, sleep disturbance, SI)	Scale of 0-3, 0=“not at all,” 3=“always.” Higher score=more severe depression	Cronbach α=0.9 (very high) [110]
	Veteran’s RAND [111] (participant self-administered)	Perceived community functioning	36 items across 8 subscales of health including physical health, limitations owing to health, energy, social health, etc	0-100, higher score=more favorable health state	Cronbach α=0.78-0.93 depending on the domain (high-very high) [112]
	Beck Suicide Scale [113] (participant self-administered)	Suicidal ideation measurement	21 items about recent thoughts of death, recent suicidal behaviors	Scale of 0-2, higher number=more severe SI	Cronbach α=0.89 (very high), strong predictive validity [114]
	PTSD Checklist for DSM-5 [115] (participant self-administered)	PTSD symptoms	20 items corresponding to DSM-5 criteria for PTSD symptoms (eg, hypervigilance, flashbacks)	Scale of 0-4, 0=not at all, 4=extremely, score >33=probable PTSD diagnosis	Cronbach α=0.94 (very high) and test-retest reliability (r_*tt*_*=*0.82) (high) [116]
	Inventory of Depressive Symptomatology, Self-Report [117] (participant self-administered)	Depression symptoms	30 items assessing DSM-5 depressive symptoms	Scale of 0-3, 0=not present, 3=severe symptoms, higher score=more severe symptoms	Cronbach α=0.94 (very high) and test-retest reliability r_*tt*_*=*0.82 (high) [118]
	Difficulties in Emotion Regulation Scale [119] (participant self-administered)	Perceived struggles with emotion modulation	36-item scale, 6 subscales of emotion regulation	Scale of 1-5, 1=almost never, 5=almost always. Higher score=more difficulty with regulation	Cronbach α=0.90 (very high) [120]
	Delayed Discounting Task [121] (administered on computer)	Impulsivity inhibition measure	Behavioral assessment of impulsivity, 27 items where participant chooses smaller immediate rewards vs larger delayed reward	Tendency to choose smaller immediate rewards consistent with more impulsivity	Test-retest reliability r_*tt*_*=*0.89 (very high) [122]
	Stroop Color & Word Test [123] (rater administered)	Impulsivity inhibition measure	Behavioral assessment of processing speed and inhibition of cognitive interference	Measure the number of correct answers versus incorrect answers	Reliability estimates range from a=0.83 to a=0.91 and test-retest reliability ranges from r_*tt*_*=*0.79 to r_*tt*_*=*0.97 (all high-very high) [124]

^a^TBI: traumatic brain injury.

^b^mTBI: mild traumatic brain injury.

^c^SI: suicidal ideation.

^d^Not applicable.

^e^UIC: University of Illinois, Chicago.

^f^CMRR: Center for Magnetic Resonance Research.

^g^MRI: magnetic resonance imaging.

^h^TMS: transcranial magnetic stimulation.

^i^PTSD: posttraumatic stress disorder.

jDSM-5: Diagnostic and Statistical Manual of Mental Disorders, Fifth Edition.

^k^AUROC: area under the receiver operating characteristic curve.

Eligibility measures and information collected during the preenrollment phone screen administered by authorized clinical researchers include The Ohio State University TBI Identification Method [92], the Columbia Suicide Severity Rating Scale [89], the UPPS-P [41], self-reported age, the ability to read and speak English, the Center for Magnetic Resonance Research safety form (Multimedia Appendix 2), and TMS safety form (Multimedia Appendix 4). If deemed eligible, participants will be asked to complete an in-person screening.

The in-person screening will consist of completing additional assessments to ensure eligibility and baseline characteristics. They include the structured diagnostic interview from the symptom attribution and classification algorithm, created by individuals involved in this study to determine likelihood and severity of prior TBIs [88], the Neurobehavioral Symptom Inventory, to determine TBI-related symptom presence [98], the Clinician-Administered PTSD Scale for DSM-5 to determine preexisting diagnosis of PTSD [99], the Alcohol Use Disorder Identification Test-Consumption Questions to determine if an alcohol use disorder is present [101], the Drug Use Disorders Identification Test to determine if a drug use disorder is present [102], and the Structured Clinical Interview for DSM-5–Research Version to determine the presence of other psychiatric disorders [104].

Once the listed diagnostic and screening measures are completed and participants meet eligibility criteria, participants will be asked to complete self-reported measures at their baseline, midpoint, and end point visits (Table 1).

Feasibility completion rates will be described according to the reasons participants did not complete the 2-week intervention and the rates of missed sessions. Study completion is defined as completing all 10 sessions. Safety measures will follow procedures used in prior TMS studies [125] and will be scored using the *Medical Dictionary for Regulatory Activities*. Side effects, AEs, and the TMS data safety sheet will assess change severity throughout the intervention. Tolerability will be measured through the number of patients who complete the treatment course and via self-report.

#### Safety and AE Monitoring

Participants will participate in safety monitoring using the Data Safety Monitoring Scale. This scale will measure rate changes from baseline vitals (temperature, blood pressure, heart rate, oxygen saturation levels), fatigue, tinnitus (ringing in the ears), sleep, dizziness, nausea, vomiting, confusion, seizure, syncope (fainting), headache, neck pain, skin integrity of the scalp, and substance use.

Participants will be asked about AEs at each iTBS session, which will be logged to ensure safety throughout the intervention. These will be coded using the current version of the Medical Dictionary for Regulatory Activities, following procedures used in previous TMS studies [82]. The TMS safety sheet (Multimedia Appendix 4) will be collected and scored regarding severity and change from baseline. Data on every side effect and AE will be collected, rated on a score of 0 to 5 to assess severity, and reported.

All iTBS sessions will be videotaped to monitor for safety and allow for review in case of an AE. Acknowledgment for pictures and videos will be completed as part of our informed consent procedure.

AEs can be nonserious and serious. A serious AE is when the changes are life-threatening and may be disabling, require hospitalization, or require intervention to prevent impairment. We will measure deleterious changes in (1) neurological status and cognitive symptoms, (2) somatic and vestibular symptoms, (3) and depression. AE will be tracked using the AE log.

All unanticipated AEs related to this research study will be reported to the IRB, the study sponsor, and the research director within 5 business days. Serious AEs will be reported to these entities within 24 hours. If any unanticipated problem occurs, such as deviation from this protocol that involves risks or has the potential to recur, the investigator will report this information to the IRB. All AE information will be reported within 2 to 5 business days of the investigator or staff becoming aware of the event.

The study will take place at the Edward Hines, Jr. VA Hospital in Hines, Illinois, a full-service, tertiary-care hospital. Our laboratory is located within the rapid response area, meaning a team of specialized nurses and doctors can be called at any time to emergently assess a patient, should the need arise. Participants will be clinically monitored for seizure by trained research staff. In the event of a seizure, the research team member will call the on-site rapid response team (RRT) to provide emergent medical care to the participant. Cardiopulmonary resuscitation–trained research staff will assess the participant’s airway, breathing, and circulation until the RRT team arrives. RRT will then take over the participant’s care, including administering seizure-abating medications and airway protection, and transport the participant to the emergency department. Any seizure will result in stopping the intervention and withdrawal from the study.

### Experimental Intervention

Each participant will be randomized to receive active or sham treatment throughout the protocol, with 50% (28/56) receiving active treatment and 50% (28/56) of participants receiving placebo. We will use a T1 MRI to localize the FP stimulation site. This region is correlated with electrode FP2 on a 10 to 20 electroencephalogram, a region overlapping with the right VMPFC [22,82]. To ensure precise and repeatable coil positioning over the frontal pole FP2 site, the Localite (neuronavigation) system and an MRI instrument marker will be used at every iTBS session.

Our 9-minute iTBS protocol will be delivered using the MagVenture Mag-Pro X100 with the MagOption stimulator, including active and placebo coils (C-B60 Butterfly coils). The consensus in the literature is that iTBS can safely deliver 1800 pulses at 110% of the MT to the frontal pole [82,83]. iTBS parameters include 3 pulses of stimulation at 50 Hz, repeated every 200 miliseconds. The interpulse interval is 20 miliseconds. A 2-second train of TBS is repeated every 10 seconds for a total of 570 seconds, equaling a total of 1800 pulses delivered over 9 minutes. To ensure that the participants can tolerate the treatment, stimulation intensity will begin at 60% to 70% of the MT intensity and will slowly increase as the patient tolerates to 110% of the patient’s MT intensity over the first few treatment sessions [42].

### Study Procedures

**Figure 2:**
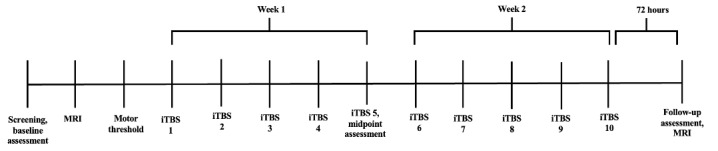
Study visit diagram. iTBS: intermittent theta burst stimulation; MRI: magnetic resonance imaging.

A partial HIPAA waiver and waiver of informed consent for screening purposes will provide regulatory approval to screen potential candidates to determine study eligibility. Once identified as a potential research candidate, candidates will be contacted for an initial phone screening to determine eligibility criteria. Demographics, mental illness diagnosis, current medication use, and history of impulsive behaviors (eg, history of fights, violence, anger, and irritability) will be cross-verified in the patient’s electronic medical record. A thorough chart review will be completed to cross-reference current medications with study eligibility criteria to identify antiepileptics or medications that could lower the seizure threshold. If review findings indicate possible contraindications to TMS or MRI related to a metal implant, then the model and manufacturer of the implant will be obtained to determine whether it is safe to expose the implant to a strong magnet. Manufacturer recommendations regarding safety will be followed. If potential participants meet all eligibility criteria, they will be invited for their first research visit, an in-person screening (visit 1). There will be 14 visits for this research study (Figure 2).

After meeting with the research staff and signing the informed consent, participants will complete several baseline diagnostic and screening measures (Table 1). These measures will be used to track changes in symptoms of impulsivity, suicidality, depression, anger, aggression, and social and occupational functioning throughout the study. If the participant is a woman, sexually active, and not on any form of physician-prescribed birth control, they will be asked to take a urine pregnancy test at this visit. Veterans who have completed baseline measures and have met eligibility criteria will be asked to complete a pretreatment resting-state fMRI at the University of Illinois, Chicago, an institution partnering with us for this study (visit 2). After the pretreatment MRI, participants will be invited back to the Hines VA to complete their MT and brain mapping using the TMS Neural Navigator (Localite) system (visit 3).

Participants will be randomized to either active or sham treatment. Blinding procedures for active and sham iTBS will be delivered with the MagProX100 with MagOption stimulator and Magpro Cool Coil B65 A/P (MagVenture), which can be switched to active or sham. The coil is identical visually for the sham and active conditions. Veterans and researchers will wear headphones connected to the noise generator during treatment to hear active iTBS for both conditions, thereby maintaining the blind. The sham coil and scalp electrodes will be placed in the same location for all participants and will mimic the sensation produced by the active coil but will not alter brain physiology. Thus, the sham stimulation looks, sounds, and feels like active stimulation. A single unblinded study team member will determine which participants are randomly placed in the active or sham groups.

After the MRI and MT have been completed, participants will be randomized, and the intervention will begin (visits 4-13). Intervention sessions will occur once daily for 10 business days, with each session lasting 30 minutes to 1 hour. Research staff will complete a TMS safety rating scale before and after every iTBS session to assess for changes from baseline for vital signs, sleep duration, fatigue level, recent seizures, and tinnitus. Pain and tolerability measures will be completed at the end of every session to assess daily pain and session tolerability. After iTBS session 5 (visit 8), each participant will be asked to complete the self-report rating scales and neurocognitive testing they completed at baseline.

All veterans who have completed the iTBS treatment course of 10 sessions will be asked to return to the University of Illinois, Chicago’s Center for Magnetic Resonance Research to obtain a second resting-state fMRI for their final research visit. Posttreatment MRI data collection is expected to be completed 72 hours after the iTBS course. This time spacing between stimulation and imaging has been done in similar studies [78] and is thought to be a long enough gap post-iTBS completion not to represent the immediate effects of the most recent stimulation.

### Analytic Plan

A statistical significance level corresponding to α=.05 will be used to test hypotheses.

Descriptive statistics of feasibility, safety, and tolerability measures will be computed for all participants and compared between active- and sham-iTBS groups. We will calculate the categorical variables’ frequencies and compare them using chi-square analyses for the descriptive statistics [126]. For the continuous variables (ie, daily pain ratings), we will compute mean, median, range, and SDs and compare using 2-tailed *t* tests.

For functional outcome measures, we will estimate a mixed model ANOVA to measure differences within-participants (how the SOFAS changes over time), between-participants (how the SOFAS differs between active and sham iTBS), and whether there is a significant interaction between time and treatment [127]. We will assume equal variance between test and control groups [128]. If the variance is unequal, we may transition to a generalized linear mixed (GLM) effects model [129].

For our neuroimaging analysis, to evaluate the VMPFC to the amygdala and anterior cingulate relationships and how they change before and after treatment, we will define ROIs for the amygdala and anterior cingulate with clear anatomical boundaries identified using Freesurfer [130]. Average time-series data will be extracted, and GLM will be used to quantify the relationship between the seeds and targets. The GLM results will yield individual *r* values (ie, correlations), which will be normalized into *Z* scores using Fisher R-to-Z transformation [131]. The individual maps of *Z* scores will be the primary measure of connectivity between the seed and target ROIs. We will then implement an ROI-to-ROI analysis within the connectivity toolbox to evaluate resting-state functional connectivity between the amygdala, anterior cingulate, and VMPFC [132]. We will assume a normal distribution and use Fisher’s *Z* test to compare active-iTBS and sham-iTBS groups, both before and after treatment [133]. We will adjust for a priori covariates, including age, sex, and baseline psychiatric diagnoses. All analyses will be corrected for multiple comparisons using false-discovery rate correction. We will use linear mixed models to test how connectivity changes correspond to functional and mental health outcome changes, with functional and behavioral measures included as the dependent variables [134].

To inform future research studies, we will estimate regression models that include additional covariates such as demographics, psychotropic medication use, comorbid psychiatric diagnoses, and scores on the Inventory of Depressive Symptomatology, Self-Report and the PTSD Checklist for DSM-5, in addition to testing our hypotheses for the primary and secondary outcomes.

## Results

This study was funded in April 2023 and began enrolling participants in July 2024. As of July 2024, there are 2 participants enrolled in the study. Data will be analyzed and study results will be disseminated on or before the project’s end date in March 2028.

## Discussion

### Principal Findings

This novel protocol represents the first pilot RCT for frontal pole iTBS for mTBI, negative urgency impulsivity, and suicidality. We hypothesize that iTBS administered to the frontal pole in US veterans with mTBI, impulsivity, and suicidality will be safe and well tolerated as studies show that both TMS and iTBS appear safe and effective when administered to other anatomical sites in individuals with mTBI [23-26]. Furthermore, studies demonstrated frontal pole iTBS is tolerable with good safety outcomes in individuals without mTBI [63].

We hypothesize that we can improve social and occupational functioning, impulsivity, and SI among US veterans with mTBI. Other studies have specifically found meaningful change in both the UPPS-P and SOFAS over a 2-week neuromodulation intervention period among individuals with major depression [90,135]. Several repetitive TMS (the precursor to iTBS) trials among individuals with SI and major depression have shown reductions in suicidality [71-74]. We anticipate these results will generalize to individuals with mTBI when treated with iTBS [72,136].

Finally, we hypothesize that we will be able to identify changes in neural connectivity among individuals who respond to our iTBS protocol by comparing pretreatment fMRIs with posttreatment fMRIs. Several studies on both iTBS and TMS, administered to the DLPFC, of a variety of treatment durations, showed increased connectivity between the frontal cortex and areas of the limbic system [137]. We expect to see similar results with iTBS administered to the VMPFC.

We will add to the existing literature in several ways. First, there are no widely established treatments for poorer functional outcomes, suicidality, or impulsivity that occur in the context of mTBI. If we can develop an effective intervention that targets all of these likely interrelated conditions, we will be adding important findings to the literature base and we could also improve the quality of life and treat SI among people with mTBI. By further establishing the frontal pole as a tolerable and effective iTBS stimulation site, we could also encourage other researchers to explore this site for potential therapeutic benefit for a variety of conditions. Finally, by identifying fMRI connectivity changes before and after iTBS treatment, we will add to the literature base regarding how neuromodulation works mechanistically, which is still unclear. Moreover, if we can identify specific fMRI connectivity changes correlating to symptomatic changes, it may help us identify future neuromodulatory treatment sites of interest, to continue to further explore this very promising treatment modality.

### Strengths of the Study

The proposed study has several key strengths. First, the study uses an established FDA-approved device readily available in both VA and civilian health care systems throughout the United States. Compared with traditional pharmacological and behavioral approaches, iTBS has a reduced latency to treatment response, which could be lifesaving among individuals with SI. Our approach also differs from previous studies that primarily focused on improving SI via the treatment of depressive symptoms. We provide a novel framework for understanding and ultimately treating SI, which could be helpful for individuals who have yet to respond to past evidence-based treatments for this condition. Finally, by targeting the VMPFC and including neuroimaging from before and after treatment, this study has the potential to identify a novel target for iTBS, increase insight into the underlying neurological mechanisms of SI, and identify which individuals are more likely to respond to a neuromodulatory intervention.

### Limitations of the Study

There are several limitations to consider. The small sample size reduces the study power, which may limit the detection of statistically significant outcome differences. However, this sample size reflects the pilot nature of the study and funding mechanism. Variability in the baseline characteristics of the patient population (ie, co-occurring psychiatric and somatic conditions, coadministration of other medications, etc) that are being studied in detail in this project may impact our findings. Covariates will be explored; however, given the small sample size, only select covariates can be added to the final models. The relatively short follow-up time of 72 hours after the final iTBS treatment will not provide insight into the long-term effects of our intervention. Future studies would benefit from additional follow-up outcomes assessments and imaging several months after the treatment to assess the intervention’s durability. Although prior studies used a dose of 110% MT and a time frame of 10 sessions, we are still determining if these parameters are optimal. A future dosing study would help optimize the treatment parameters. This study does not select targets based on resting-state data; however, in collecting these data in tandem using neuronavigation, we can potentially target more specific sites in the future.

### Conclusions

The proposed study integrates past findings from impulsivity, TBI, neuroanatomical, neuroimaging, neurorehabilitation, and neurostimulation research to create a novel, but highly informed treatment strategy. In this study, we hope to induce neuroplasticity in the connections between the VMPFC and limbic system, through neuromodulation at the frontal pole. In inducing neuroplasticity with iTBS, in an effort to repair damaged neural connections, we hope to mitigate the development of impulsive behaviors, improve social and community functioning, and decrease SI, as we believe all 3 of these conditions would benefit from strengthening the VMPFC-limbic system network. Data obtained by using validated scales will inform future studies.

Moreover, the collection of pretreatment neuroimaging may help us understand what neural signatures predispose individuals to respond favorably to frontal pole iTBS, so we might be able to predict who is most likely to benefit from treatment, following comparable work in predicting response to neurostimulation in individuals with major depression [138]. Thus, findings from the proposed project are expected to have the capacity to (1) help identify which individuals are more likely to respond to a neurostimulation intervention for impulsivity and suicidality related to mTBI and (2) better understand what neural changes we are eliciting. Advancing knowledge in both areas will inform future studies and enable a more rapid determination of which treatments will be most effective for a given individual and determining where connectivity changes are occurring may allow us to further optimize treatment effects.

## Appendix

Multimedia Appendix 1 CONSORT (Consolidated Standards of Reporting Trials) flow diagram—anticipated.

Multimedia Appendix 2 University of Illinois–Chicago Center for Magentic Resonance Research magnetic resonance imaging safety form.

Multimedia Appendix 3 Equipment list.

Multimedia Appendix 4 Transcranial magnetic stimulation (TMS) safety form.

## Abbreviations

ABP: abductor pollicis brevis
AE: adverse event
CONSORT: Consolidated Standards of Reporting Trials
DLPFC: dorsolateral prefrontal cortex
FDA: Food and Drug Administration
fMRI: functional magnetic resonance imaging
FP: frontal polar cortex
GLM: generalized linear mixed
HIPAA: Health Insurance Portability and Accountability Act
IRB: institutional review board
iTBS: intermittent theta burst stimulation
MEP: motor-evoked potential
MRI: magnetic resonance imaging
MT: motor threshold
mTBI: mild traumatic brain injury
PTSD: posttraumatic stress disorder
ROI: region of interest
RRT: rapid response team
SA: suicide attempt
SI: suicidal ideation
SOFAS: Social and Occupational Functioning Assessment Scale
SPIRIT: Standard Protocol Items: Recommendations for Interventional Trials
TBI: traumatic brain injury
TMS: transcranial magnetic stimulation
UPPS-P: Urgency, Premeditation (lack of), Perseveration (lack of), Sensation Seeking, Positive Urgency Impulsive Behavior Scale–Negative Urgency subscale VA: US Veterans Administration
VMPFC: ventromedial prefrontal cortex

## Data Availability

The datasets generated during and analyzed during this study are not publicly available due to legal restrictions the US Department of Veterans Affairs places on access to veteran data, including both identifying data and sensitive patient information, but are available from the corresponding author on reasonable request with the completion of an approved data use agreement. For more information, please visit the VA website [139].
