# Supplementary material for: Frontal Pole Neuromodulation for Impulsivity and Suicidality in Veterans With Mild Traumatic Brain Injury and Common Co-Occurring Mental Health Conditions: Protocol for a Pilot Randomized Controlled Trial
Source: JMIR Res Protoc. 2024 Dec 13;13:e58206. doi: 10.2196/58206 (PMC11681286; doi:10.2196/58206)
Supplement: Multimedia Appendix 1 [file resprot_v13i1e58206_app1.doc]

**Figure 3**: **CONSORT Flow Diagram**. Anticipated

**Enrollment**

Informed consent obtained & in- person eligibility screening completed (n=56)

Screened for eligibility

Randomization (n=56)

Excluded: Not enrolled

♦  Not meeting initial inclusion criteria

♦ Participant withdrawal

**Intervention**

**Follow-Up**

**Analysis**

**Allocation**

Excluded

♦  Not meeting complete inclusion criteria

♦ Participant withdrawal

Lost to follow-up

Received intervention

♦ Intervention completers

♦ Intervention non-completers

Primary and secondary outcome analysis
♦ All participants who complete at least one iTBS session will be included in safety, feasibility and tolerability analyses

Active (n=28)

Sham (n=28)

Dropout

♦  Relocation

♦ Withdrawal

♦ Unable to tolerate intervention

♦ Etc

Dropout

♦  Relocation

♦ Withdrawal

♦ Unable to tolerate intervention

♦ Etc
