# Supplementary material for: Frontal Pole Neuromodulation for Impulsivity and Suicidality in Veterans With Mild Traumatic Brain Injury and Common Co-Occurring Mental Health Conditions: Protocol for a Pilot Randomized Controlled Trial
Source: JMIR Res Protoc. 2024 Dec 13;13:e58206. doi: 10.2196/58206 (PMC11681286; doi:10.2196/58206)
Supplement: Multimedia Appendix 2 [file resprot_v13i1e58206_app2.docx]

**Center for MR Research**

Research Subject Assessment

**Name** Click or tap here to enter text.

**Weight** Click or tap here to enter text.

**Consent obtained (indicate with signature subject/investigator)? X** Click or tap here to enter text.

**Please answer the medical history questions below:**

Are you left handed or right handed? Click or tap here to enter text.

|  | Yes |  | No |  |  | Yes |  | No |  |
| --- | --- | --- | --- | --- | --- | --- | --- | --- | --- |
| Aneurysm clip |  |  |  |  | Tattoos/Piercing |  |  |  |  |
| Pacemaker/stimulator |  |  |  |  | Cardiac/Respiratory problem |  |  |  |  |
| Ear Implant |  |  |  |  | Strokes/Seizures |  |  |  |  |
| Heart Valve Replaced |  |  |  |  | Liver/Kidney disease |  |  |  |  |
| Other Implant |  |  |  |  | Cancer |  |  |  |  |
| History of Metal Work |  |  |  |  | Brain Surgery |  |  |  |  |
| Hx of Metal in eyes |  |  |  |  | Diabetes/High BP |  |  |  |  |
| Bullets/Shrapnel |  |  |  |  | Claustrophobia |  |  |  |  |
| Dentures/Braces/Wigs |  |  |  |  | Prior MRI: yes  no  Where: Click or tap here to enter text. | | | | |

Onset of Symptoms (date/time) Click or tap here to enter text.

Expanded Medical History Click or tap here to enter text.

Current Meds Click or tap here to enter text.

AllergiesClick or tap here to enter text. Contrast to be given? Yes  No

Pregnant? Yes  No  Unsure

Source of Screening Info. Click or tap here to enter text.Screened by Click or tap here to enter text.

xClick or tap here to enter text. Click or tap here to enter text.

Signature of Consent Date
