# Supplementary material for: Frontal Pole Neuromodulation for Impulsivity and Suicidality in Veterans With Mild Traumatic Brain Injury and Common Co-Occurring Mental Health Conditions: Protocol for a Pilot Randomized Controlled Trial
Source: JMIR Res Protoc. 2024 Dec 13;13:e58206. doi: 10.2196/58206 (PMC11681286; doi:10.2196/58206)
Supplement: Multimedia Appendix 3 [file resprot_v13i1e58206_app3.docx]

**Equipment List**

GE Discover MR750 3.0T MRI Scanner

Localite TMS Neural Navigator System with Localite Neural Navigator Software (v 3.3.22) pre-installed. System consists of:

-Desktop Computer

-Localite optical tracking camera

-Trackers for TMS coils and subject

MagVenture MagPro X100 TMS Machine with MagOption Simulator, along with:

-C-B60 coil for motor threshold

-Active/Sham self-cooling coil (Cool Coil B65 A/P)

-Coil cooling system

**Neuroimaging Software**

SPM12 (Statistical Parametric Mapping, Wellcome Trust Center for Neuroimaging, Cambridge, UK)

CONN Toolbox v. 22a (Neuroimaging Tools and Resources Collaboratory, MIT, Cambridge, MA)

Freesurfer v. 7.4.1 (MGH, Harvard, Cambridge, MA)
