# Supplementary material for: Frontal Pole Neuromodulation for Impulsivity and Suicidality in Veterans With Mild Traumatic Brain Injury and Common Co-Occurring Mental Health Conditions: Protocol for a Pilot Randomized Controlled Trial
Source: JMIR Res Protoc. 2024 Dec 13;13:e58206. doi: 10.2196/58206 (PMC11681286; doi:10.2196/58206)
Supplement: Multimedia Appendix 4 [file resprot_v13i1e58206_app4.docx]

**Transcranial Magnetic Stimulation Safety Checklist**

| **Yes** | **No** | **Please check ‘yes’ or ‘no’ if the following items apply:** |
| --- | --- | --- |
|  |  | Cardiac Pacemaker |
|  |  | Implanted Cardiac Defibrillator |
|  |  | Take anti-epileptic medication |
|  |  | Seizure within the last 3 months |
|  |  | Family history of seizure/epilepsy |
|  |  | Unexplained Seizures |
|  |  | History of stroke |
|  |  | Implanted Medical Pump |
|  |  | Serious Heart Disease |
|  |  | Increased Intracranial Pressure |
|  |  | Pregnant |
|  |  | Take tricyclic anti-depressants such as amitryptiline, nortriptyline |
|  |  | Take neuroleptic medications such as Seroquel, Geodon, olanzapine |
|  |  | Any secondary conditions that may alter electrolyte balance – like kidney disease or autoimmune disease |
|  |  | Implanted conductive, ferromagnetic, or other magnetic-sensitive metals in the head (e.g., cochlear implants, aneurism clips or coils, stents, bullet fragments) |

Checklist Items collected from Wasserman 1998 NINDS Guidelines (*Electroencephalography and clinical Neurophysiology* 108:1-16) and Neuronetics NeuroStar® TMS System User’s Manual.

**If you marked yes to any of the following, please explain/provide details below:**
